# Hemin suppresses interferon responses in periodontal cell models through JAK2–STAT1 inhibition

**DOI:** 10.3389/fimmu.2026.1958476

**Published:** 2026-09-30

**Authors:** Xiaoyu Huang, Reinhard Gruber, Layla Panahipour

**Affiliations:** 1 Department of Oral Biology, Medical University of Vienna, Vienna, Austria; 2 Department of Prosthodontics, The Affiliated Stomatological Hospital of Southwest Medical University, Luzhou, China; 3 Department of Periodontology, School of Dental Medicine, University of Bern, Bern, Switzerland

**Keywords:** CXCL10 (C-X-C motif ligand 10), hemin, HSC2, interferon signaling, JAK - STAT signaling pathway, periodontal fibroblasts, periodontitis

## Abstract

**Introduction:**

Hemin, an oxidized heme species released during erythrocyte breakdown, is detectable in gingival crevicular fluid during periodontal inflammation, but its effects on local immune signaling remain unclear. We investigated whether hemin modifies cytokine-induced interferon responses in HSC-2 oral squamous cell carcinoma cells and primary human gingival fibroblasts.

**Methods:**

Cells were stimulated with interleukin-1β and tumor necrosis factor-α, or with interferon-γ, in the presence or absence of hemin. Transcriptomic changes were assessed by RNA sequencing and pathway analysis, and selected transcripts and proteins were validated by reverse-transcription quantitative PCR, enzyme-linked immunosorbent assay, immunoblotting, and immunofluorescence. Ligand–kinase interactions were examined by molecular docking and molecular dynamics simulations, and recombinant Janus kinase 2 activity was measured in a cell-free assay.

**Results:**

Hemin preferentially suppressed an interferon-associated gene network, including CXCL10, CXCL11, MX1, OAS1, IFIT1, IFIT3, and ISG15, while nuclear factor κB-associated responses were comparatively preserved. Hemin also reduced signal transducer and activator of transcription 1 Tyr701 phosphorylation and nuclear accumulation induced by either the cytokine combination or interferon-γ. Computational analyses predicted stable hemin interactions within conserved ATP-binding pockets of Janus kinases, and the cell-free assay demonstrated inhibition of recombinant Janus kinase 2.

**Discussion:**

Together, these findings show that hemin attenuates interferon-associated transcription in periodontal cell models, consistent with inhibition of the Janus kinase 2–signal transducer and activator of transcription 1 axis. This mechanism may enable blood-derived hemin to modify local mucosal immune signaling, although its relevance to periodontal tissues and antiviral defense requires confirmation in organotypic, infection, and in vivo models.

## 1 Introduction

Periodontitis remains a major global public-health burden and a leading cause of destruction of the tooth-supporting tissues (1–3). Among the clinical parameters used to assess periodontal health and disease, bleeding on probing is one of the most widely applied indicators of gingival inflammation and disease activity (4–8). Traditionally, periodontal bleeding has been interpreted mainly as a consequence of inflammation, epithelial ulceration, and vascular disruption (9, 10). However, recurrent micro-bleeding may also actively modify the periodontal microenvironment by exposing epithelial and stromal cells to blood-derived molecules that are normally absent or present only at low concentrations in the gingival sulcus (11–13).

Free heme accumulates in injured and inflamed tissues as a consequence of erythrocyte lysis and hemoglobin degradation (14). Rather than serving only as a marker of tissue damage, extracellular heme is increasingly recognized as a bioactive regulator of oxidative stress, innate immunity, inflammation, and wound biology (14, 15). In periodontitis, free heme has been detected in gingival crevicular fluid, with approximately 20-fold higher concentrations at diseased than healthy sites and associations with clinical periodontal parameters (16). Because chronic periodontal lesions are characterized by ulcerated pocket epithelium, inflammatory exudation, and recurrent bleeding, resident periodontal cells are likely exposed to repeated hemin stimulation (17). Whether this exposure actively modulates inflammatory signaling in periodontal tissues remains insufficiently understood.

Hemin is best known as a potent inducer of the nuclear factor erythroid 2-related factor 2 (NRF2)–heme oxygenase 1 (HMOX1) stress-response pathway (18). Through NRF2 activation, hemin induces HMOX1 and downstream cytoprotective responses that support cellular adaptation to oxidative and inflammatory stress (19, 20). However, hemin may also regulate inflammation through mechanisms that are not solely explained by secondary HMOX1 activity. Of particular relevance, heme can interact with protein kinase domains, and structural studies have identified heme-binding motifs within the catalytic domain of JAK2 (21). As signal transducer and activator of transcription 1 (STAT1) activation requires upstream JAK activity, and STAT1 signaling has been linked to inflammatory bone loss in experimental periodontitis (22), JAK–STAT signaling represents a plausible molecular target through which hemin may shape oral immunity.

Canonical inflammatory responses are largely regulated by NF-κB and MAPK signaling, whereas interferon-associated responses depend on Janus kinase–signal transducer and activator of transcription signaling, particularly the JAK–STAT1 axis (23, 24). CXCL10 and CXCL11 are established STAT1-dependent chemokines that mediate immune-cell recruitment and have been detected in periodontal tissues and oral mucosal inflammatory lesions (25–27). In addition, the JAK–STAT pathway is central to epithelial antiviral defense by mediating interferon-induced expression of antiviral genes, and viral infections have been implicated as potential modifiers of periodontitis pathogenesis (28, 29). Thus, JAK–STAT signaling links antiviral host defense, chemokine-driven immune-cell recruitment, and chronic oral inflammation.

Here, we tested whether hemin suppresses cytokine-induced interferon signaling in periodontal cell models and examined the JAK2–STAT1 axis as a candidate mechanism. We combined transcriptomics with targeted validation, imaging, computational ligand–kinase analysis, and a cell-free JAK2 activity assay. The study was designed to identify a molecular effect of hemin under defined cell-culture conditions; it did not test infection or tissue-level outcomes.

## 2 Materials and methods

### 2.1 Cell culture and treatments

Human oral squamous cell carcinoma (HSC-2) cells, originally obtained from the Health Science Research Resources Bank (Sennan, Japan), were cultured in Dulbecco’s Modified Eagle Medium (DMEM; Sigma-Aldrich, St. Louis, MO, USA) supplemented with 10% fetal bovine serum (FBS; Bio&Sell GmbH, Nuremberg, Germany) and a 1% antibiotic-antimycotic (Sigma-Aldrich, St. Louis, MO, USA) solution. Primary human gingival fibroblasts were isolated from three healthy donors and maintained in DMEM containing 10% FBS and 1% penicillin/streptomycin (Sigma-Aldrich, St. Louis, MO, USA). Tissue collection protocols were approved by the Ethics Committee of the Medical University of Vienna (EK NR 631/2007), and written informed consent was obtained from all donors.

To evaluate cytotoxicity and determine optimal dosing, cells were seeded at a density of 3 × 10^4^ cells/cm^2^ in 96-well plates and exposed to hemin (#T5515; TargetMol, Boston, MA, USA) under serum-free conditions (37 °C, 5% CO_2_). For the MTT assay, cells were treated with serial dilutions of hemin (ranging up to 50 μM) for 24 h, after which they were incubated with 0.5 mg/mL MTT solution (Sigma-Aldrich, St. Louis, MO, USA) for 2 h at 37 °C. Following medium aspiration, the resulting formazan crystals were dissolved in 50 *μ*L of dimethyl sulfoxide (DMSO; Sigma-Aldrich, St. Louis, MO, USA), and absorbance was measured at 570 nm using a Synergy HTX multi-mode microplate reader (BioTek Instruments, Winooski, VT, USA). In parallel, for the Live/Dead assay, live and dead cell populations were visualized via green and red fluorescence, respectively, after treatment with targeted hemin concentrations (0, 10, 20, and 40 μM) for 9 h using a Live/Dead Cytotoxicity Assay Kit (#ALX-850-249; Enzo Life Sciences, Farmingdale, NY, USA).

Based on these viability profiles, a working concentration of 10 μM hemin was established for subsequent experiments. Following 2 h of hemin stimulation, HSC-2 cells were fixed by microwave-assisted dual fixation (2.5% glutaraldehyde, 2% paraformaldehyde in 0.1 M sodium cacodylate buffer, pH 7.4) using a Pelco BioWave Pro with SteadyTemp Pro (three 2-min pulses at 150 W, 21 °C, with 2-min pauses). After washing (4 × 10 min in cacodylate buffer), cells were postfixed in 1% osmium tetroxide (1 h), washed again (4 × 10 min), dehydrated through a graded ethanol series (30–100%, 10 min per step) and pure acetone (2 × 10 min), and embedded in Epon 812 resin (Serva; 40 °C for 24 h, polymerized at 60 °C for 48 h). Ultrathin sections (~50 nm) were cut on a Leica UC7 ultramicrotome, mounted on copper mesh grids, and contrasted with 1% uranyl acetate (7 min) and 3% lead citrate (3 min). Images were acquired on a Tecnai G2–20 transmission electron microscope (TEM; operated at 80 kV) with an FEI Eagle 4K CCD camera at the Center for Anatomy and Cell Biology, Medical University of Vienna. Contrast was equalized (CLAHE) and adjusted for brightness/contrast in Fiji (30).

To model cytokine-induced inflammation, cells were stimulated with recombinant human IL-1β and TNF-α (10 ng/mL each; ProSpec-Tany TechnoGene Ltd., Rehovot, Israel) in the presence or absence of 10 μM hemin. To assess the influence of physiological protein buffering on hemin efficacy, control experiments were performed using medium supplemented with 10% pooled human serum (#H5667, Sigma-Aldrich, Saint Louis, MO, USA)—providing endogenous albumin and hemopexin—during the stimulation period. Additionally, recombinant human Interferon-γ (IFN-γ; 100 ng/mL; Thermo Fisher Scientific, Bremen, Germany) was utilized specifically to assess canonical STAT1 signaling activation. The CO-releasing molecule-3 (CORM-3; #T6452, TargeMol, Boston, MA, USA) and biliverdin (hydrochloride; #19257, Cayman Chemical, Ann Arbor, MI, USA) were employed in parallel to evaluate whether these two principal catabolites of heme degradation were sufficient to reproduce the suppressive effect of hemin on chemokine or IFN-induced pSTAT1 nuclear translocation. N-acetyl-L-cysteine (NAC; #A9165, Sigma-Aldrich, Saint Louis, MO, USA) and tin (IV) protoporphyrin IX chloride (SnPP; #16375, Cayman Chemical, Ann Arbor, MI, USA) were used as pharmacological tools to assess the contribution of reactive oxygen species and HO-1 catalytic activity, respectively, to hemin-mediated suppression of *CXCL10* and *CXCL11*. HSC-2 cells were pretreated with NAC (2 mM) for 2 h or SnPP (10 μM) before hemin treatment, or treated with CORM-3 (50 μM), biliverdin (10 μM), followed by cytokine stimulation as described above. Gene expression was assessed by RT-qPCR, while CXCL10 protein levels in the culture supernatant from the CORM-3 exploratory screening were quantified by ELISA.

### 2.2 RNA sequencing and bioinformatics

Following a 9 h treatment, total RNA was extracted from HSC-2 cells using the Gene MATRIX Universal RNA Purification Kit (EURx, Gdańsk, Poland) in conjunction with an rDNase set (Macherey-Nagel, Düren, Germany). Sequencing libraries were prepared at the Core Facility Genomics of the Medical University of Vienna using the QuantSeq 3’ mRNA-Seq Library Prep Kit FWD for Illumina (Lexogen) with unique dual indices. Based on initial qPCR assessments, library amplification was performed using 15 PCR cycles. Library quality and insert size were verified on a Bioanalyzer 2100 (Agilent) using a High Sensitivity DNA Kit, and quantification was performed via the Qubit dsDNA HS Assay (Invitrogen). Pooled libraries were sequenced on an Illumina NextSeq 2000 instrument using a P3 flow cell in a 1×75 bp single-end mode, yielding an average of 12 million reads per sample.

Raw reads in FASTQ format were demultiplexed using the Illumina bcl2fastq tool (v2.19.1.403) and the Lexogen idemux tool. Read trimming and filtering were performed using cutadapt (v2.8) to remove poly-A tails, ambiguous bases (N’s), and low-quality bases (Phred score < 30) from the 3’ ends. Following quality control (retaining an average of 8.7 million reads per sample), trimmed reads were aligned to the human reference genome (GRCh38) using Gencode 29 annotations via the STAR aligner (v2.6.1a) in 2-pass mode. Raw read counts per gene were quantified by STAR (31), and differential gene expression analysis was executed using the DESeq2 package (v1.40.2) (32) in R, employing the lfcShrink (33) method for fold-change estimation.

### 2.3 Transcriptomic and pathway analysis

All computational and statistical analyses were performed in the R environment (v4.5.0; r-project.org). Principal component analysis (PCA) was executed using the prcomp function, with the first two principal components visualized via *ggplot2*. Differentially expressed genes were visualized as heatmaps (*p*_adj_ < 0.05, |log_2_FC| ≥ 1) and volcano plots (*p*adj < 0.01, |log_2_FC| ≥ 1.5). Gene Set Enrichment Analysis (GSEA) and GO/KEGG pathway functional enrichments were subsequently conducted, with strategies prioritizing downregulated differentially expressed genes (DEGs) to identify pathogenic signaling circuits suppressed by hemin, while upregulated DEGs were analyzed to capture intrinsic protective stress responses and heme/iron metabolic adaptation. Protein-protein interaction (PPI) data were retrieved from the STRING database, and the resulting networks were subsequently visualized in the R environment.

### 2.4 ChIP-seq data visualization and motif mapping

To visualize STAT1 chromatin binding enrichment, standardized human signal density tracks (https://www.encodeproject.org/, Experiment ID: ENCSR000EZK; cell type: HeLa-S3) were retrieved from the ENCODE Portal and uniformly aligned to the hg38 reference genome assembly. Genomic coordinates and gene models were mapped based on the GENCODE registry (Release 49). High-resolution track visualization was generated using the *Gviz* package (v1.46.0). The visualization window was restricted to a 20-kb regulatory region flanking the *CXCL10* locus (chr4:76,935,000–76,955,000), with a vertical alignment guide programmatically inserted at chr4:76,941,500 to register the peak summit relative to the canonical transcription start site (TSS). To further confirm sequence-level occupancy, the scanned nucleotide topology within this anchored core promoter zone was cross-referenced and validated against the standard STAT1 canonical binding motif (Matrix ID: MA0137.4, Class: STAT domain factors) retrieved from the open-access JASPAR 2026 Core Database (http://jaspar.genereg.net/).

### 2.5 qPCR and ELISA

RNA extraction methods were as described previously. RNA concentration and purity were evaluated using a NanoDrop 1000 Spectrophotometer (Thermo Fisher Scientific, Bremen, Germany). Only high-quality RNA samples (A260/280 ≥ 1.6) were used for downstream applications. Reverse transcription and subsequent quantitative real-time PCR were performed using LabQ reagents (Labconsulting, Vienna, Austria) on a CFX Connect™ Real-Time PCR Detection System (Bio-Rad Laboratories, Hercules, CA, USA). Target gene expression levels were calculated using the ΔΔCt method (34) and normalized to the housekeeping gene *GAPDH*. All primer sequences are listed in the **Supplementary Table 1**. To evaluate the translational impact on the CXCR3-chemokine axis, as the primary representative effector molecule, secreted CXCL10 protein levels in the culture supernatants of HSC-2 cells and gingival fibroblasts were quantified using a human CXCL10/IP-10 DuoSet ELISA kit (#DY226; R&D Systems, Minneapolis, MN, USA) according to the manufacturer’s instructions.

### 2.6 Immunofluorescence staining

Cells were seeded at a density of 15,000 cells/cm² on Millicell^®^ EZ slides (Merck KGaA, Darmstadt, Germany). Following pretreatment with hemin for 15 min, cells were stimulated with either IL-1β/TNF-α for 3 h or IFN-γ for 45 min. Samples were then fixed in 4% paraformaldehyde, permeabilized with 100% methanol (Carl Roth, Karlsruhe, Germany), and blocked with 5% normal goat serum (#5425; Cell Signaling Technology, Danvers, MA, USA). Cells were probed with a rabbit anti-pSTAT1 primary antibody (#9167, 1:400; Cell Signaling Technology, Danvers, MA, USA), followed by an Alexa Fluor^®^ 488-conjugated anti-rabbit secondary antibody (#4412, 1:1000; Cell Signaling Technology, Danvers, MA, USA). Fluorescence images were captured using an Echo Revolve hybrid microscope.

### 2.7 Immunoblotting

Cells cultured at a density of 30,000 cells/cm² were lysed in 1X RIPA Lysis Buffer, diluted from a 10X stock solution (#20-188; EMD Millipore, Billerica, MA, USA), supplemented with cOmplete™ ULTRA protease and PhosSTOP™ phosphatase inhibitor cocktails (Roche). Equal amounts of protein lysates were resolved by SDS-PAGE and transferred onto 0.45 μm nitrocellulose membranes (Amersham Protran Premium, GE Healthcare Life Science, Germany). Membranes were probed with rabbit anti-phospho-STAT1 and anti-STAT1 primary antibodies (#9167 and #9172, respectively; both at 1:1000; Cell Signaling Technology) and subsequently incubated with an HRP-conjugated anti-rabbit IgG secondary antibody (#7074, 1:10,000; Cell Signaling Technology). To verify equal protein loading, membranes were incubated with an HRP-conjugated mouse monoclonal anti-β-actin antibody (#sc-47778 HRP, 1:1000; Santa Cruz Biotechnology). Protein bands were visualized using Clarity™ Western ECL Substrate (Bio-Rad Laboratories, Hercules, CA, USA). and captured on a ChemiDoc Imaging System (Bio-Rad Laboratories, Hercules, CA, USA).

### 2.8 ROS detection

Intracellular ROS levels were assessed using the fluorescent probe H2DCFH-DA (MedChemExpress, Monmouth Junction, NJ, USA). HSC-2 cells were seeded in 96-well black plates, allowed to adhere overnight, and treated with 10 µM hemin for 2 h. Following treatment, cells were washed with PBS, incubated with 10 µM DCFH-DA in serum-free medium at 37 °C for 20 min in the dark, and washed again to remove excess probe, then imaged using an Echo Revolve hybrid microscope.

### 2.9 Molecular docking

AutoDock Vina (v1.2.5) (35, 36) was utilized to dock hemin (https://pubchem.ncbi.nlm.nih.gov/, PubChem CID: 455658) (37)against the ATP-binding pockets of JAK1 (PDB ID: 3EYG), JAK2 (PDB ID: 3E63), and JAK3 (PDB ID: 3LXL), downloaded from the RCSB Protein Data Bank (https://www.rcsb.org/) in PDB format (38). Promising docking poses were subjected to 100-ns all-atom molecular dynamics (MD) simulations in GROMACS 2025 on a workstation featuring an i7-13700K/32GB/RTX 4070/Win11 Pro. Protein topologies were parameterized with AMBER99SB-ILDN (JAK1/2) or AMBER14SB (JAK3) force fields; hemin was parameterized with GAFF2 via ACPYPE. Complexes were solvated in TIP3P water (1.2 nm clearance, 0.15 M NaCl) and neutralized.

### 2.10 Molecular dynamics

Following 2 ns equilibration phases under both NVT (constant volume) and NPT (constant pressure) ensembles, 100-ns production runs were executed at 310 K and 1 bar (2 fs time step). GROMACS utilities were used to compute root mean square deviation (RMSD), root mean square fluctuation (RMSF), and hydrogen bond occupancies. Thermodynamics and binding free energies (with per-residue decomposition) were calculated over the final 50 ns using the MM-PBSA method. Free energy landscapes (FEL) were mapped via PCA-based Gibbs free energy analysis.

### 2.11 JAK2 kinase activity assay

Direct enzymatic inhibition of JAK2 was evaluated using a commercial JAK2 Assay Kit (BPS Bioscience, San Diego, CA, USA). Briefly, recombinant human JAK2 was incubated with ATP (10 µM final) and a Poly (Glu: Tyr, 4:1) substrate in the presence of hemin (5 µM), or reference inhibitor Staurosporine (10 nM; Sigma-Aldrich, St. Louis, MO, USA) at 30 °C for 45 min. Remaining ATP levels, inversely proportional to kinase activity, were quantified using the Kinase-Glo^®^ Luminescent Kinase Assay (Promega, Madison, WI, USA). Luminescence was recorded on a microplate reader (BioTek Instruments, Winooski, VT, USA). Percentage inhibition was calculated as (RLU~test~ − RLU~PC~)/(RLU~Blank~ − RLU~PC~) × 100%.

### 2.12 Statistical analysis

All *in vitro* experiments were conducted in at least three independent biological replicates. Statistical comparisons between treatment groups and corresponding positive or negative controls were performed utilizing the ratio-paired t-test. All data compilation and statistical analyses were executed in GraphPad Prism version 9 (GraphPad Software, La Jolla, CA, USA). The statistical significance was set at *p* < 0.05.

## 3 Results

### 3.1 Hemin cytocompatibility and working concentration

MTT analysis and live/dead staining showed that 10 μM hemin was well tolerated by HSC-2 cells and primary gingival fibroblasts, whereas higher concentrations reduced metabolic activity (**Supplementary Figures 1A–D**). TEM confirmed that hemin-treated HSC-2 cells maintained intact nuclear membranes, mitochondria, and preserved cytoplasmic ultrastructure, excluding cytotoxic injury as a confounding factor (**Supplementary Figure 1E**). Based on these findings, 10 μM hemin was selected for all subsequent mechanistic experiments.

### 3.2 Hemin reshapes the cytokine-induced transcriptome

RNA sequencing was performed to determine how hemin modifies the epithelial response to IL-1β/TNF-α. Principal component analysis showed clear separation of untreated, hemin-treated, cytokine-treated, and cytokine-plus-hemin-treated cells, indicating that each condition produced a distinct transcriptional state (**Figure 1A**). Hierarchical clustering confirmed that IL-1β/TNF-α induced a broad inflammatory transcriptional program and that hemin cotreatment altered a defined subset of cytokine-responsive genes (**Figure 1B**). Hemin alone induced a limited but distinct stress-response signature, including *HMOX1*, *STC2*, *GCLM*, *SLC7A11*, *PTGS2*, and *FOSL1* (**Figure 1C**; **Supplementary Figure 2**; **Supplementary Table 2**). IL-1β/TNF-α induced a broad inflammatory response, whereas cotreatment with hemin reduced the number of upregulated genes and increased the number of downregulated genes (**Figures 1D, E**; **Supplementary Table 2**). Thus, hemin reshaped the cytokine-induced transcriptome rather than globally suppressing inflammatory transcription.

**Figure 1:**
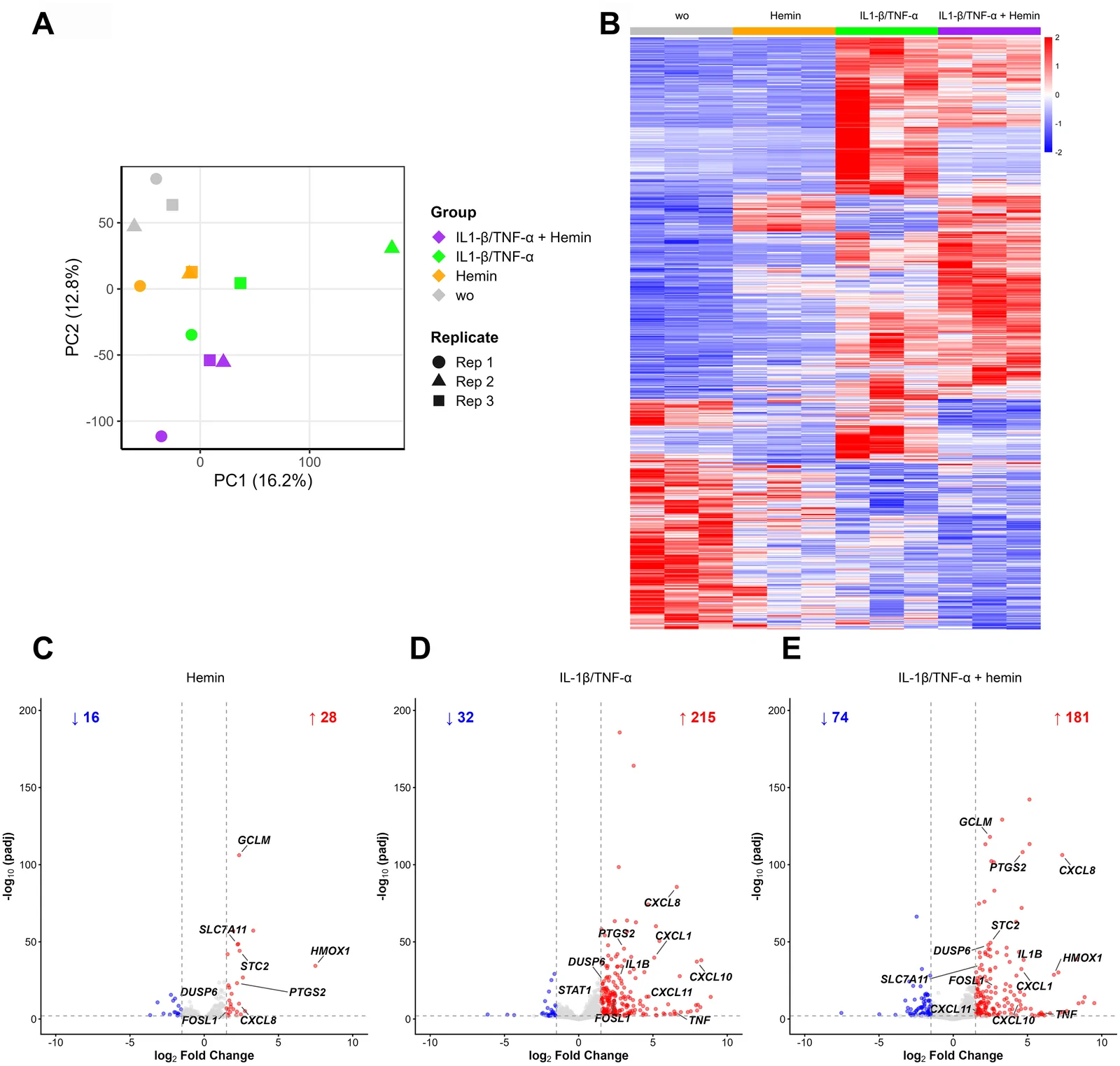
Hemin reshapes the cytokine-induced transcriptome of oral epithelial cells. HSC-2 cells were either untreated or stimulated for 9 h with hemin, IL-1β/TNF-α, or IL-1β/TNF-α plus hemin before bulk RNA sequencing. **(A)** Principal component analysis (PCA) showing separation of treatment groups based on global gene expression. Each point represents one biological replicate. **(B)** Heat map of differentially expressed genes (DEGs) across treatment groups (*p*_adj_ < 0.05, |log_2_FC| ≥ 1). Rows represent genes and columns represent individual samples; expression values are shown as row-scaled z-scores. **(C–E)** Volcano plots of genome-wide DEGs (*p*_adj_ < 0.01, |log_2_FC| ≥ 1.5) in HSC-2 cells treated with hemin alone **(C)**, IL-1β/TNF-α alone **(D)**, or the combination **(E)**. These genes represent the results of an unbiased whole-transcriptome RNA-seq analysis, rather than a pre-filtered inflammation-related gene set. Red and blue dots indicate upregulated and downregulated genes; selected genes are labeled. Hemin alone induced a discrete transcriptional program (28 up, 16 down) dominated by HMOX1 and stress-response genes. IL-1β/TNF-α produced a broad inflammatory response (215 up, 32 down). Hemin cotreatment selectively attenuated interferon-associated genes while largely preserving NF-κB-driven transcription (181 up, 74 down).

### 3.3 Hemin preferentially suppresses interferon-associated genes

To identify the cytokine-induced pathways affected by hemin, we directly compared IL-1β/TNF-α plus hemin with IL-1β/TNF-α alone. This comparison identified 74 downregulated and 17 upregulated genes (**Figure 2A**; **Supplementary Table 3**). The downregulated genes were dominated by interferon-stimulated genes, including *CXCL10*, *CXCL11*, *MX1*, *OAS1*, *IFIT1*, *IFIT3*, *STAT1*, and *ISG15*. Gene set enrichment analysis (GSEA) showed significant negative enrichment of IFN-α and IFN-γ signaling pathways (**Figure 2B**), whereas reactive oxygen species (ROS) and NF-κB signaling remained positively enriched (**Figure 2C**). Further GO and KEGG pathway analyses linked the hemin-suppressed gene set to antiviral defense and innate immune signaling pathways, and STRING network analysis confirmed that most downregulated genes formed a densely connected interferon-associated module (**Figures 2D–F**), indicating that hemin preferentially suppresses interferon-associated transcription while preserving other inflammatory and stress-response pathways.

**Figure 2:**
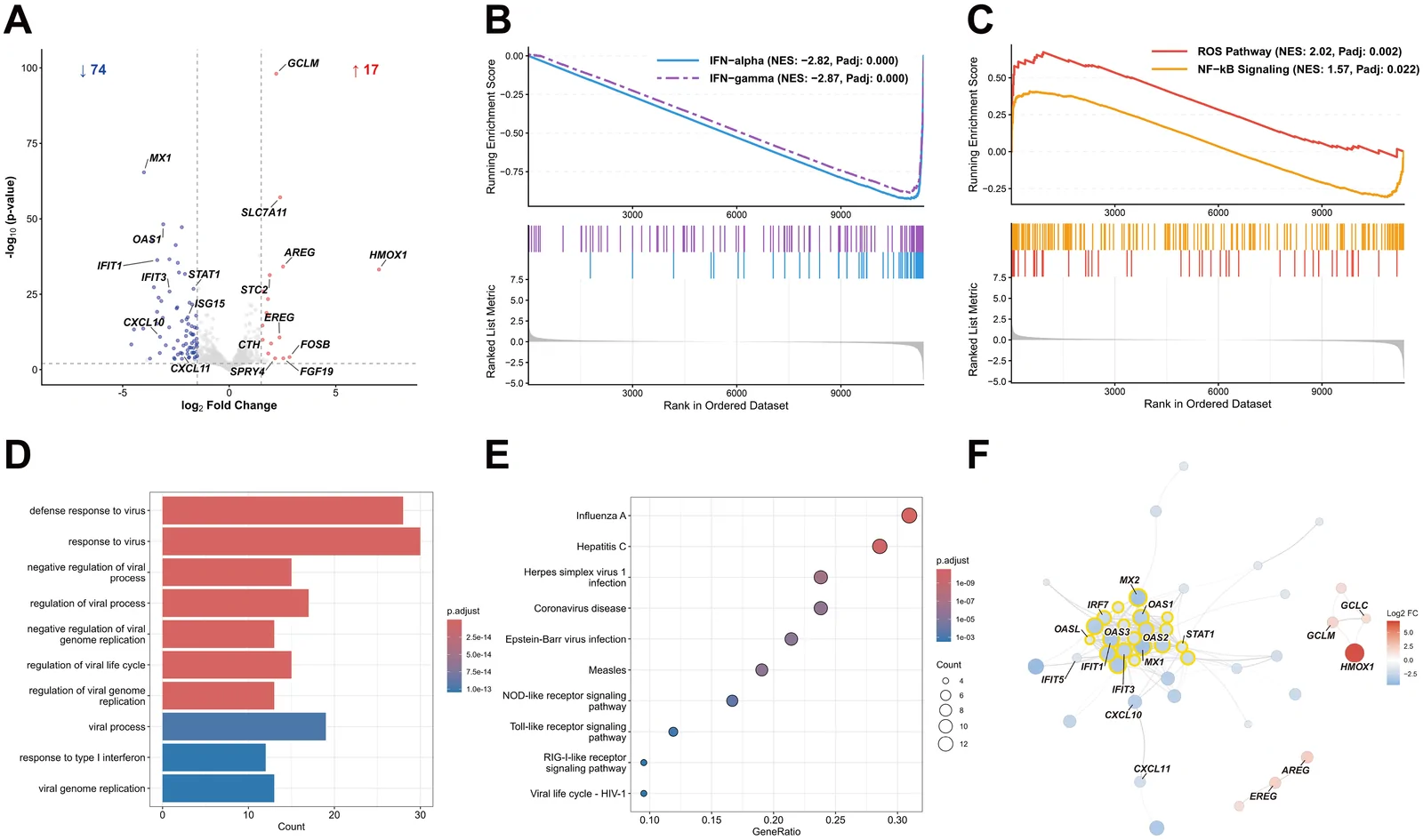
Hemin selectively suppresses an interferon-associated gene network. Differential expression analysis was performed by comparing HSC-2 cells stimulated with IL-1β/TNF-α plus hemin against cells stimulated with IL-1β/TNF-α alone. **(A)** Volcano plot showing genes altered by hemin under IL-1β/TNF-α-stimulated conditions (17 up, 74 down; *p*_adj_ < 0.01, |log_2_FC| ≥ 1.5). Downregulated cytokine-induced genes are highlighted, and selected interferon-associated genes are labeled. **(B, C)** Gene Set Enrichment Analysis (GSEA) demonstrating significant negative enrichment of IFN-α (solid blue line; NES = −2.82, Padj < 0.001) and IFN-γ (dashed purple line; NES = −2.67, Padj < 0.001) signaling pathways **(B)** and positive enrichment of ROS (red line; NES = 2.02, Padj = 0.002) pathway and NF-κB (orange line; NES = 1.57, Padj = 0.022) signaling pathway **(C)**. The upper panel shows running enrichment scores; the lower panel displays the ranked list metric and gene hit positions within the ordered dataset. **(D)** Gene Ontology (GO) biological process enrichment analysis of downregulated DEGs, showing top enriched terms related to antiviral defense and the type I interferon response, ranked by gene count. Bar color indicates adjusted p-value. **(E)** KEGG pathway enrichment analysis of downregulated DEGs, showing top-enriched pathways, predominantly related to viral infection and innate immune signaling (RIG-I-like, NOD-like, and Toll-like receptor signaling pathways). Dot size indicates gene count; dot color indicates adjusted p-value; Gene Ratio denotes the proportion of input genes annotated to each pathway. **(F)** STRING protein–protein interaction network of differentially expressed genes following hemin treatment. Node color reflects log_2_ fold change (red, upregulated; blue, downregulated); node size reflects the degree of connectivity. The densely interconnected cluster (highlighted) comprises canonical interferon-stimulated genes, whereas upregulated genes (HMOX1, GCLC, GCLM) are peripherally distributed. Edge thickness indicates the confidence of interaction. FC, fold change.

### 3.4 Hemin suppresses CXCL10 and CXCL11 in periodontal cells

To validate the transcriptomic findings, RT-qPCR and ELISA were performed. In HSC-2 cells, IL-1β/TNF-α strongly induced *CXCL10*, *CXCL11*, *CXCL1*, and *CXCL8*. Hemin consistently reduced *CXCL10* and *CXCL11* transcript levels and *CXCL10* protein release (**Figures 3A, B**), whereas its effects on *CXCL1* and *CXCL8* were inconsistent. This pattern was replicated in primary gingival fibroblasts (**Figures 3C, D**). Furthermore, HSC-2 cells stimulated with IL-1β/TNF-α were treated with hemin under serum-free or 10% human serum (HS) conditions: hemin robustly suppressed cytokine-induced *CXCL10* and *CXCL11* expression without serum, whereas 10% HS — providing physiological hemin-binding proteins (albumin, hemopexin) — markedly attenuated hemin bioavailability and decreased its anti-inflammatory efficacy (**Figure 3E**). These findings indicate that serum protein buffering is a critical determinant of hemin bioactivity, potentially limiting the translational relevance of acute high-concentration hemin exposure observed under protein-free conditions. Consistent with broader attenuation of interferon-associated transcription, hemin also reduced induction of the classical interferon-stimulated genes *MX1*, *MX2*, and *OAS1* in HSC-2 cells (**Supplementary Figure 3A**). Hemin likewise reduced IFN-γ-induced expression of these chemokines and interferon-stimulated genes in gingival fibroblasts (**Supplementary Figure 3B**), supporting a shared effect across the two periodontal cell models.

**Figure 3:**
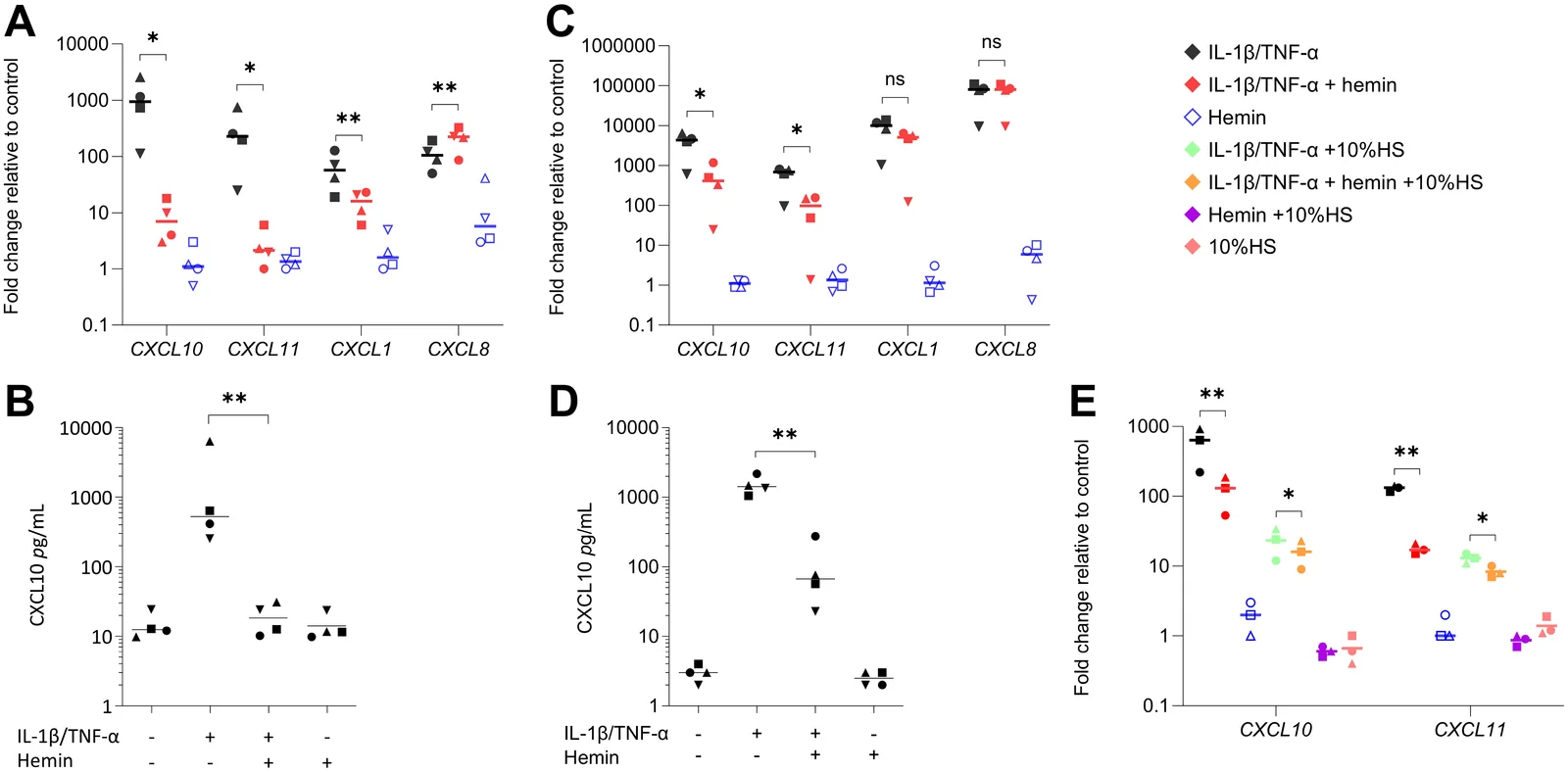
Hemin suppresses interferon-associated chemokines in epithelial cells and gingival fibroblasts. HSC-2 cells and gingival fibroblasts were stimulated with IL-1β/TNF-α with or without hemin, and chemokine expression was assessed by RT-qPCR and ELISA. **(A)** RT-qPCR analysis of cytokine-induced chemokine expression in HSC-2 cells. Hemin reduced *CXCL10* and *CXCL11*, whereas effects on *CXCL1* and *CXCL8* were less consistent. **(B)** CXCL10 protein release from HSC-2 cells was measured by ELISA. **(C)** RT-qPCR analysis of chemokine expression in gingival fibroblasts. Hemin reduced *CXCL10* and *CXCL11*, whereas *CXCL1* and *CXCL8* were comparatively preserved. **(D)** CXCL10 protein release from gingival fibroblasts was measured by ELISA. Data are expressed as fold change relative to untreated controls for RT-qPCR and as pg/mL for ELISA. **(E)** Effect of human serum protein-buffering on hemin-mediated suppression of inflammatory chemokines in HSC-2 cells. HSC-2 cells were stimulated with IL-1β/TNF-α in the presence or absence of hemin under serum-free or 10% human serum (HS) conditions. Hemin robustly suppressed cytokine-induced *CXCL10* and *CXCL11* expression without serum. In the presence of 10% HS, physiological protein buffering (human serum albumin, hemopexin) markedly attenuated hemin bioavailability, resulting in a loss of significant anti-inflammatory efficacy. Symbols indicate independent experiments. Statistical comparisons were performed with ratio-paired t tests. *p < 0.05; **p < 0.01; ns, not significant.

### 3.5 Suppression of CXCL10 and CXCL11 is independent of ROS and HMOX1 activity

Because hemin strongly induced HMOX1 and heme-adaptive genes, we examined whether oxidative stress, HMOX1 activity, or carbon monoxide signaling accounted for the suppression of interferon-associated chemokines. Hemin did not significantly increase intracellular ROS under the experimental conditions used (**Supplementary Figure 4A**). Moreover, scavenging cytosolic free radicals with N-acetylcysteine (NAC) did not restore cytokine-induced *CXCL10* or *CXCL11* expression in the presence of hemin (**Supplementary Figure 4B**). Pharmacologic inhibition of HMOX1 with Tin (IV) Protoporphyrin IX Chloride (SnPP) also failed to prevent hemin-mediated suppression of *CXCL10* and *CXCL11* (**Supplementary Figure 4C**). Biliverdin, in contrast to hemin, did not reproduce the suppressive effect on IFN-γ-induced nuclear phospho-STAT1 accumulation in either cell type (**Supplementary Figure 4D**). Likewise, carbon monoxide release by CORM-3 did not reproduce or substantially modify the suppressive effect of hemin on supernatant CXCL10 protein levels (**Supplementary Table 4**). These findings indicate that although hemin activates a robust HMOX1 stress-response program, the suppression of CXCL10, CXCL11, and other ISGs is not primarily explained by ROS scavenging, carbon monoxide signaling, or biliverdin production.

### 3.6 Hemin impairs STAT1 activation

Network analysis identified STAT1 as a highly connected hub among the interferon-stimulated genes affected by hemin. Because CXCL10 and CXCL11 are classical STAT1-dependent chemokines, we next examined whether hemin interferes with STAT1 activation. Public STAT1 ChIP-seq data supported STAT1 occupancy at the *CXCL10* regulatory region (**Supplementary Figure 5**). IL-1β/TNF-α slightly increased STAT1 Tyr701 phosphorylation in HSC-2 cells. IFN-γ served as a canonical positive control. Hemin pretreatment strongly reduced STAT1 phosphorylation induced by either IL-1β/TNF-α or IFN-γ (**Figure 4A**). Immunofluorescence microscopy confirmed reduced phosphorylated STAT1 signal and impaired nuclear accumulation in both HSC-2 cells and gingival fibroblasts (**Figure 4B**). In contrast, p65 nuclear translocation and p65/p38 phosphorylation were largely preserved (**Supplementary Figure 6**). These findings identify STAT1 activation as a selective signaling checkpoint affected by hemin.

**Figure 4:**
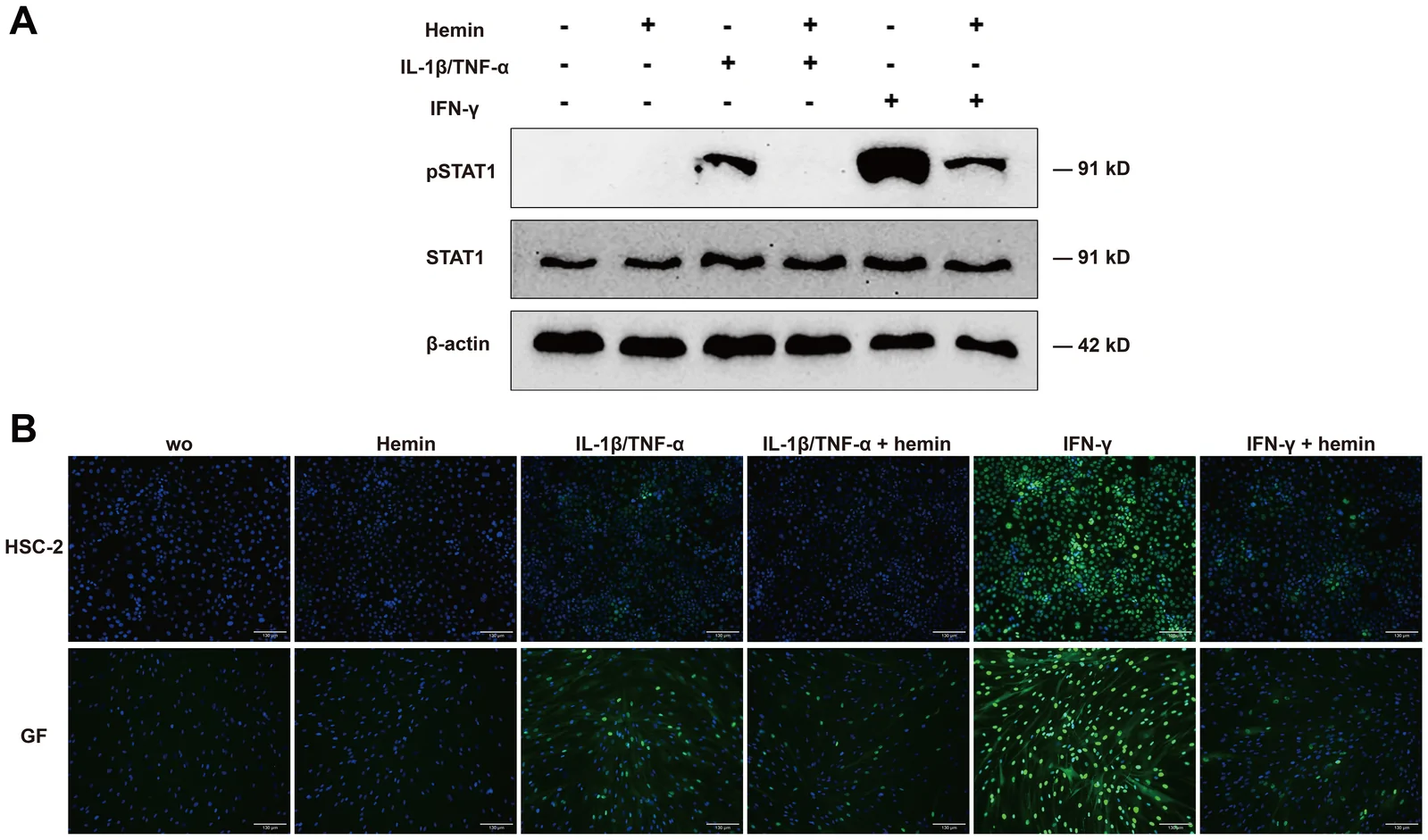
Hemin impairs STAT1 phosphorylation and nuclear accumulation. Cells were pretreated with hemin for 15 min and then stimulated with IL-1β/TNF-α for 3 h or IFN-γ for 45 min to assess STAT1 activation. **(A)** Representative western blot of phosphorylated STAT1 Tyr701, total STAT1, and β-actin in HSC-2 cells. IL-1β/TNF-α and IFN-γ induced STAT1 phosphorylation, whereas hemin reduced STAT1 phosphorylation without reducing total STAT1. **(B)** Representative merged immunofluorescence images of HSC-2 cells and gingival fibroblasts (GF) stained for phosphorylated STAT1 (green) and nuclei (DAPI, blue). IL-1β/TNF-α and IFN-γ promoted nuclear accumulation of phosphorylated STAT1, whereas hemin reduced the phosphorylated STAT1 signal and impaired nuclear localization. Scale bars, 130 μm.

### 3.7 Hemin directly inhibits JAK2 kinase activity

To determine whether hemin could target upstream JAKs, we performed molecular docking, predicting favorable binding of hemin to JAK1, JAK2, and JAK3 within conserved ATP-binding pockets, with key interactions including hydrogen bonds with Asp1003, Arg1007, and Asn1008 (JAK1); hydrophobic contacts with Leu855, Asp939, and Arg980 and a hydrogen bond to Leu932 (JAK2); and hydrogen bonds with Arg953, Lys855, and Asn832 plus additional hydrophobic contacts (JAK3) (**Figure 5A**). 100-ns molecular dynamics (MD) simulations confirmed complex stability, with root-mean-square deviation (RMSD) values plateauing at approximately 10 ns (**Figure 5B**). Molecular Mechanics Poisson–Boltzmann Surface Area (MM-PBSA) analysis yielded binding free energies (ΔG) of −18.7, −25.2, and −25.6 kcal/mol for JAK1, JAK2, and JAK3, respectively, with per-residue energy decomposition identifying Val889, Leu1010, and Gly882 (JAK1); Leu983, Gly856, and Val863 (JAK2); and Cys909, Leu956, and Val836 (JAK3) as the residues contributing most favorably to binding (**Figure 5C**). Extended MD analyses—including radius of gyration (Rg), per-residue root-mean-square fluctuation (RMSF), solvent-accessible surface area (SASA), hydrogen-bond counts, thermodynamic energy components, and Gibbs free energy landscape (FEL)—further confirmed stable folding, sustained protein–ligand interactions, van der Waals–dominated binding, and stable, low-energy conformational basins across all 3 hemin–JAK complexes (**Figures 6A–F**). In a cell-free JAK2 kinase activity assay, hemin (5 µM) inhibited recombinant JAK2 activity by more than 60%, comparable to staurosporine (10 nM; positive control; ns, ratio-paired *t* test) (**Figure 5D**). Together with the observed reduction in STAT1 phosphorylation and nuclear accumulation, these findings are consistent with a mechanism involving direct JAK2 inhibition.

**Figure 5:**
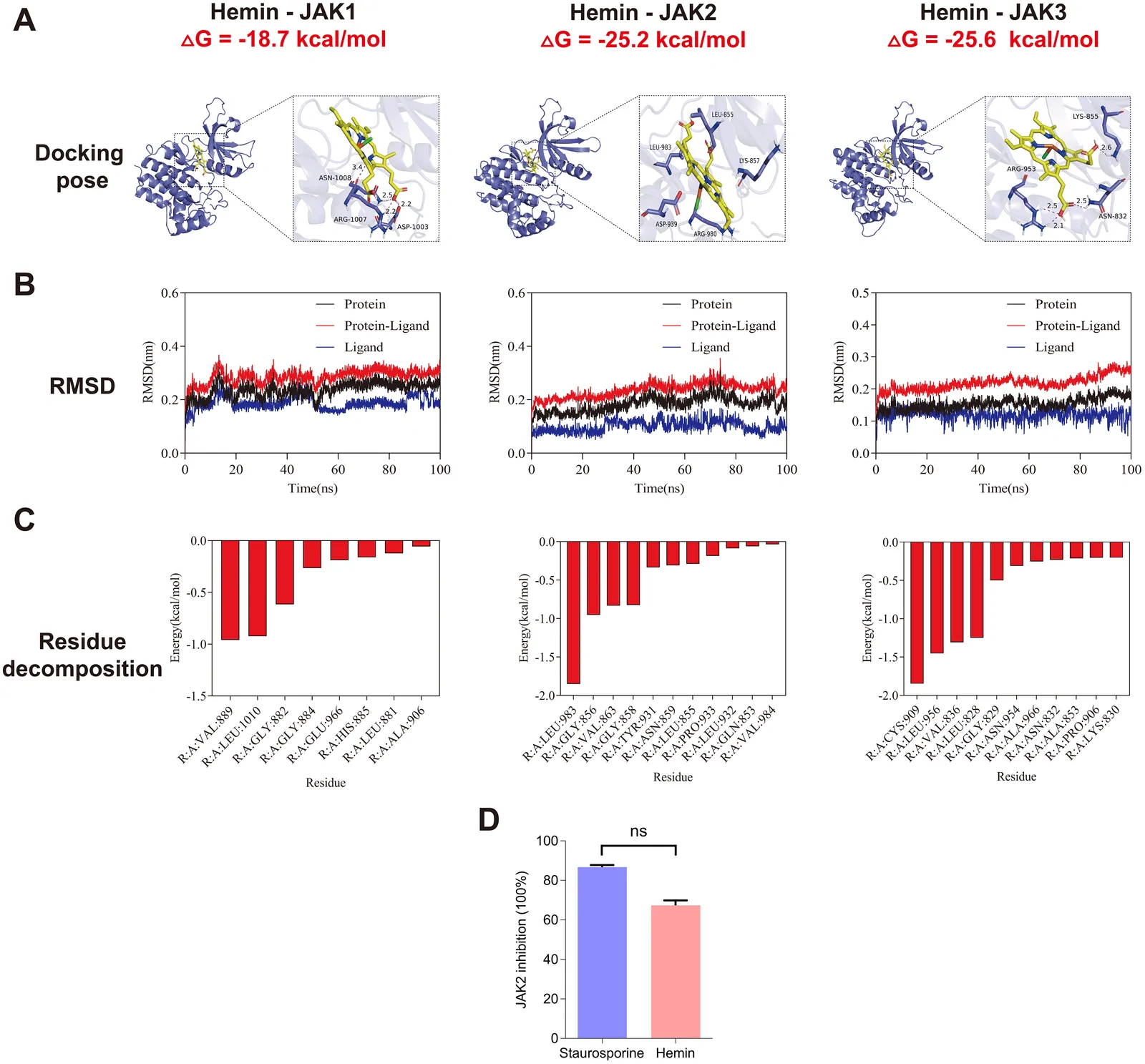
Computational analyses and a cell-free assay support direct inhibition of JAK2 by hemin. Molecular docking and 100-ns molecular dynamics (MD) simulations were used to evaluate the interactions of hemin with JAK1, JAK2, and JAK3. **(A)** Representative docking poses of hemin (yellow sticks) within the ATP-binding pockets of JAK1, JAK2, and JAK3. Key interacting residues are labeled: hydrogen bonds with Asp1003, Arg1007, and Asn1008 in JAK1; hydrophobic contacts with Leu855, Asp939, and Arg980 and a hydrogen bond with Leu932 in JAK2; and hydrogen bonds with Arg953, Lys855, and Asn832 plus additional hydrophobic contacts in JAK3. Hydrogen bonds are shown as dashed lines. **(B)** Root-mean-square deviation (RMSD) of the protein backbone and hemin over the 100-ns MD trajectory. RMSD values stabilized after approximately 10 ns, indicating stable complex formation for all 3 JAK–hemin complexes. **(C)** Per-residue binding free energy decomposition derived from Molecular Mechanics Poisson–Boltzmann Surface Area (MM-PBSA) calculations. Bars represent the energy contribution (kcal/mol) of individual residues to the total binding free energy. Residues with the most favorable contributions include Val889, Leu1010, and Gly882 (JAK1); Leu983, Gly856, and Val863 (JAK2); and Cys909, Leu956, and Val836 (JAK3). Overall binding free-energy values (ΔG: −18.7, −25.2, and −25.6 kcal/mol for JAK1, JAK2, and JAK3, respectively) are indicated above each panel. **(D)** JAK2 kinase inhibition by hemin in a cell-free luminescence-based assay. JAK2 kinase activity was measured in the presence of hemin (5 µM) or staurosporine (10 nM; positive control). Inhibition is expressed as a percentage relative to the vehicle control. Data are presented as mean ± SD; *n* = 3 independent experiments. The bracket compares hemin with staurosporine; ns, not significant (ratio-paired *t-test*). The JAK1 and JAK3 results are computational predictions and do not establish direct enzymatic inhibition of these isoforms.

**Figure 6:**
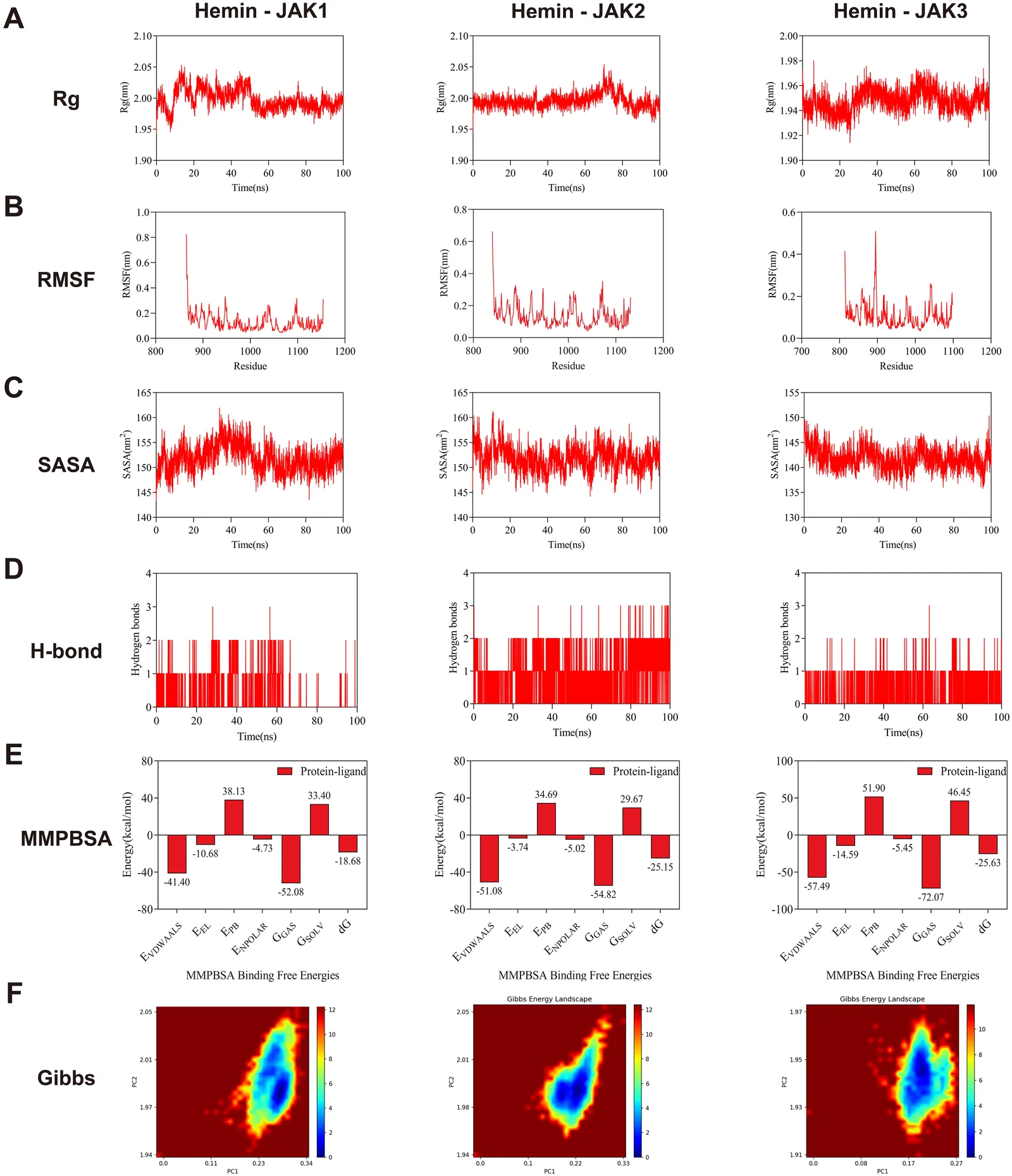
Extended molecular dynamics analyses confirm the stability of hemin–JAK complexes. Additional 100-ns MD simulation metrics for hemin bound to JAK1, JAK2, and JAK3. **(A)** Radius of gyration (Rg) of the hemin–JAK complexes throughout the simulation, indicating overall structural compactness. Rg values showed no sustained increasing or decreasing trends, confirming stable folding. **(B)** Per-residue root-mean-square fluctuation (RMSF) profiles of JAK1, JAK2, and JAK3 in complex with hemin, reflecting local backbone flexibility. Most residues exhibited low RMSF values, with higher fluctuations localized to the N-termini. **(C)** Solvent-accessible surface area (SASA) of the hemin–JAK complexes over the simulation period, confirming stable surface exposure and intact hydrophobic cores. **(D)** Hydrogen-bond analysis between hemin and key residues of JAK1, JAK2, and JAK3 throughout the 100-ns trajectory. Hydrogen-bond counts stabilized after approximately 10 ns, indicating sustained protein–ligand interactions. **(E)** MM-PBSA binding free-energy components—van der Waals, electrostatic, polar solvation, and nonpolar solvation—for the hemin–JAK1, –JAK2, and –JAK3 complexes, illustrating the thermodynamic basis of binding. **(F)** Gibbs free energy landscape (FEL) constructed from principal component analysis (PC1 and PC2), illustrating the dominant conformational states sampled during the simulation. Deep-blue basins correspond to high-probability conformational clusters representing stable states; yellow/red regions indicate transition states or energy barriers. FEL, free energy landscape; MM-PBSA, Molecular Mechanics Poisson–Boltzmann Surface Area; ns, not significant; Rg, radius of gyration; RMSD, root-mean-square deviation; RMSF, root-mean-square fluctuation; SASA, solvent-accessible surface area.

## 4 Discussion

This study provides evidence that hemin can attenuate interferon-associated signaling in periodontal cell models. In HSC-2 oral epithelial cells and primary gingival fibroblasts, hemin reduced responses linked to the JAK2–STAT1 axis, a pathway central to interferon-driven cytokine and host-defense programs (39–41). Rather than producing broad anti-inflammatory suppression, hemin reduced STAT1-dependent chemokines, most prominently CXCL10 and CXCL11, while nuclear factor κB-associated responses were comparatively preserved. In a cell-free assay, hemin directly inhibited recombinant JAK2 activity, consistent with evidence that heme can bind and modulate protein tyrosine kinases, including JAK2 (21, 42). Together, these data link hemin exposure under defined *in vitro* conditions to reduced STAT1 phosphorylation, impaired nuclear accumulation of phosphorylated STAT1, and suppressed interferon-associated gene expression. They raise the possibility that blood-derived hemin released during periodontal bleeding could modify local mucosal immune signaling, a hypothesis that requires validation in tissue-level and *in vivo* models.

Transcriptomic analysis showed that hemin preferentially suppressed an interferon-stimulated gene network, including *CXCL10*, *CXCL11*, *MX1*, *MX2*, *OAS1*, *IFIT1*, *IFIT3*, *STAT1*, and *ISG15*. These genes are typical components of type I and type III interferon-induced antiviral and inflammatory programs (24, 29, 41, 43, 44). Importantly, this was not a global shutdown of inflammatory activation. NF-κB-associated and oxidative stress pathways remained active, indicating that hemin alters the quality rather than the overall magnitude of inflammation. This interpretation was reinforced by validation experiments in both HSC-2 cells and primary gingival fibroblasts, in which hemin reduced *CXCL10* and *CXCL11* expression and reduced CXCL10 protein release, whereas *CXCL1* and *CXCL8* were comparatively preserved. Thus, the validated chemokine profile argues against nonspecific toxicity and supports pathway-specific regulation of STAT1-dependent inflammatory signaling.

CXCL10 is a central validated readout in this study. As a prototypic interferon-inducible chemokine, CXCL10 links STAT1 activation to recruitment of activated T lymphocytes, natural killer cells, and monocytes through CXCR3-dependent signaling (45, 46). Its suppression at the transcriptomic, messenger RNA, and protein levels supports attenuation of a biologically relevant STAT1-dependent inflammatory pathway. Together with reduced *CXCL11* expression, this pattern could influence CXCR3-positive immune-cell recruitment in periodontal tissues. However, immune-cell migration and tissue-level recruitment were not measured, so this interpretation remains a hypothesis.

Beyond its anti-inflammatory implications, the broader transcriptomic pattern raises a second and potentially more far-reaching hypothesis. Hemin suppressed not only *CXCL10* and *CXCL11* but also classical interferon-stimulated antiviral genes, including *MX1*, *MX2*, *IFIT1*, *IFIT3*, *OAS1*, and *ISG15*, which constitute core components of epithelial antiviral defense downstream of JAK–STAT signaling (24, 29, 43). This observation is particularly intriguing because impaired antiviral immunity is a well-recognized consequence of pharmacologic JAK inhibition (47). Patients receiving JAK inhibitors consistently exhibit an increased incidence of viral infections, especially herpes zoster, reflecting attenuation of interferon-dependent host defense (48, 49).

These observations raise the possibility that hemin exposure could attenuate epithelial antiviral programs and produce a transcriptional phenotype resembling aspects of pharmacologic JAK inhibition. However, the present study measured neither viral infection nor antiviral activity. It therefore does not show impaired viral control, persistence, or reactivation, and it does not establish that viruses initiate or perpetuate periodontitis. These questions require direct testing in oral epithelial infection models.

Hemin strongly induced HMOX1 and other heme-adaptive genes, consistent with activation of the NRF2–HMOX1 pathway, the principal cytoprotective response to heme and oxidative stress (19, 20). However, inhibition of HO-1 activity, antioxidant treatment, and carbon monoxide donation did not explain the suppression of *CXCL10* and *CXCL11*. While previous reports (50) indicated that heme oxygenase 1 can negatively regulate IFN signaling over longer timeframes via autophagy-mediated IRF3/7 degradation, our findings demonstrate that parent hemin acts as a zero-delay structural pan-JAK competitor. This direct physical occlusion of the JAK catalytic pocket provides immediate mucosal protection prior to the onset of secondary metabolic programs. These findings suggest that although the NRF2–HMOX1 pathway is robustly activated by hemin, it is not the principal mechanism responsible for inhibition of interferon-associated signaling. A key mechanistic observation was direct inhibition of recombinant JAK2 by hemin in the cell-free assay. Molecular docking and molecular dynamics simulations predicted stable hemin interactions within ATP-binding pockets of JAK family kinases. Together, these results provide a candidate explanation for the rapid reduction in STAT1 Tyr701 phosphorylation and impaired nuclear accumulation after IL-1β/TNF-α or IFN-γ stimulation.

JAK–STAT signaling is central to inflammatory, autoimmune, and antiviral responses, and pharmacologic JAK inhibitors are established therapeutic agents (39, 51). The present results raise the possibility that hemin released after tissue bleeding could attenuate JAK2–STAT1 signaling, although physiological relevance will depend on the amount and binding state of locally bioavailable hemin. In acute wounds, transient modulation of interferon signaling may influence resolution and tissue repair (52). In chronic inflammatory lesions, sustained exposure to extracellular heme may modify host responses while other inflammatory pathways remain active (20, 53). Together, these observations support investigation of hemin as a context-dependent regulator of cytokine signaling after bleeding.

This study has several limitations. First, mechanistic experiments relied primarily on HSC-2 carcinoma cells in monolayer culture; while primary human gingival fibroblasts confirmed key endpoints, neither model fully recapitulates the stratified oral epithelium. Second, a single 10 μM hemin dose and a short stimulation period did not allow assessment of dose–response relationships or delayed interferon signaling. Free haem in GCF is higher at diseased than healthy periodontal sites (mean 1.12 μM vs 0.047 μM) (16). Therefore, 10 μM hemin should be viewed as an acute *in vitro* mechanistic stimulus rather than a steady-state model of GCF exposure. *In vivo*, albumin and hemopexin bind free hemin, further limiting direct quantitative extrapolation from *in vitro* dosing to GCF dynamics (54, 55). Third, the study design excluded microbial challenge, viral infection, organotypic tissue models, and *in vivo* systems. Fourth, direct enzymatic inhibition was confirmed only for JAK2, whereas JAK1 and JAK3 interactions remain computationally predicted. Fifth, although CXCL10 and CXCL11 both exhibited coordinated transcriptional suppression, protein-level verification was limited to CXCL10; CXCL11 secretion kinetics require further investigation. Finally, transcriptional downregulation of MX1, MX2, and other interferon-stimulated genes was not assessed in functional viral infection models. Consequently, these data do not directly establish impaired antiviral defense, altered viral persistence, or clinical translational outcomes of hemin-mediated JAK2 inhibition in human periodontal tissues.

Future studies should test whether hemin-mediated suppression of interferon-stimulated genes translates into impaired epithelial antiviral defense. Oral epithelial infection models using interferon-sensitive viruses, such as herpes simplex virus type 1, could determine whether reduced MX1, OAS1, IFIT, ISG15, CXCL10, and CXCL11 expression increases viral replication or alters antiviral surveillance (43). Such experiments would distinguish transcriptional suppression from a functional antiviral phenotype and clarify whether recurrent periodontal bleeding affects local host defense beyond inflammation.

## 5 Conclusion

Hemin attenuated STAT1-dependent interferon responses in HSC-2 cells and primary gingival fibroblasts, with CXCL10 as a validated transcript and protein readout. Direct inhibition of recombinant JAK2 in a cell-free assay provides a candidate mechanism for reduced STAT1 phosphorylation and nuclear accumulation. These findings identify a molecular effect of hemin on cytokine signaling in periodontal cell models and support further investigation of blood-derived hemin as a modifier of mucosal immunity. Tissue-level, infection, and *in vivo* studies are required to establish physiological relevance.

## Data Availability

The raw sequencing data generated in this study have been deposited in the NCBI Gene Expression Omnibus (GEO) and are accessible under accession number GSE335283.
