# Supplementary material for: Hemin suppresses interferon responses in periodontal cell models through JAK2–STAT1 inhibition
Source: Front Immunol. 2026 Sep 30;17:1958476. doi: 10.3389/fimmu.2026.1958476 (PMC13661988; doi:10.3389/fimmu.2026.1958476)
Supplement: Supplementary file 1 [file DataSheet1.zip › Corrected_Supplementary_Materials/Supplementary materials.pdf]

Title: Hemin suppresses interferon responses in periodontal cell models through JAK2–STAT1 inhibition

Xiaoyu Huang [1,2], Reinhard Gruber [1,3*], Layla Panahipour[1]

[1] Department of Oral Biology, Medical University of Vienna, 1090 Vienna, Austria
[2] Department of Prosthodontics, The Affiliated Stomatological Hospital of Southwest Medical University, Luzhou, 646000, China
[3] Department of Periodontology, School of Dental Medicine, University of Bern, 3010 Bern, Switzerland

Correspondence: reinhard.gruber@meduniwien.ac.at; Tel: + 43 1 40070 2660

Supplementary Files

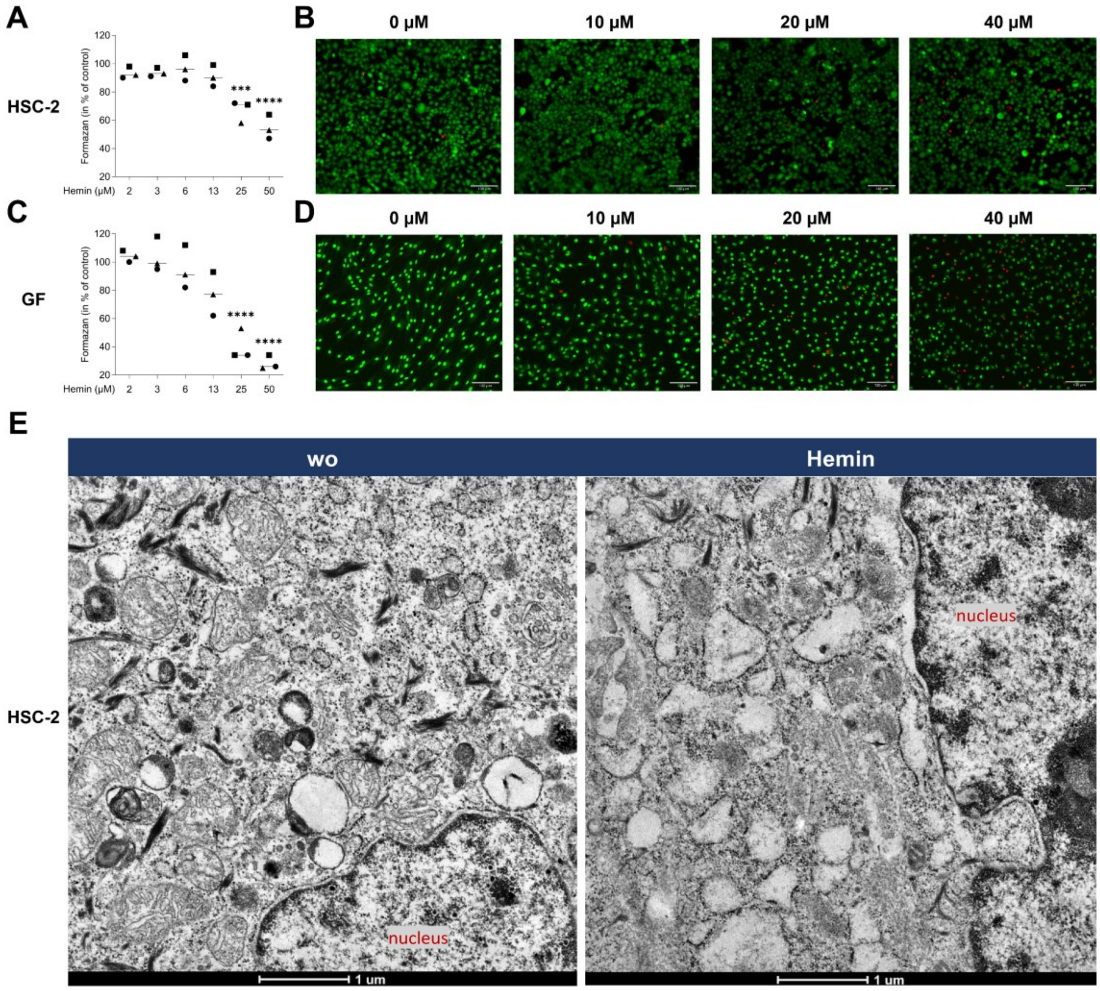

Supplementary Figure 1. Hemin cytocompatibility in HSC-2 cells and gingival fibroblasts. HSC-2 cells and gingival fibroblasts were exposed to increasing concentrations of hemin under serum-free conditions. **(A, C)** MTT analysis of HSC-2 cells and gingival fibroblasts (GF) after 24 h. ***$p < 0.001$; ****$p < 0.0001$. **(B, D)** Representative live/dead staining after 9 h, with live cells and dead cells indicated by the staining channels used in the assay. Scale bars: 130 µm. **(E)** Transmission electron microscopy (TEM) of HSC-2 cells treated with 10 µM hemin for 2 h, showing preserved ultrastructure. Wo, untreated cells. Scale bars, 1 µm. These data supported the use of 10 µM hemin for mechanistic experiments.

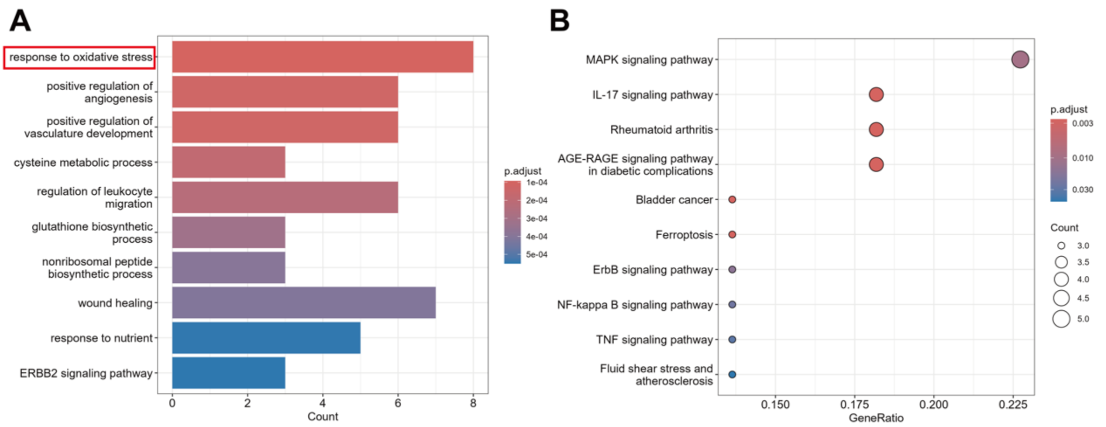

Supplementary Figure 2. Transcriptional profiling of hemin-treated HSC-2 cells reveals a heme-adaptive and oxidative stress response signature.

Bioinformatic analysis of genes regulated by hemin alone in HSC-2 cells. **(A)** Gene Ontology (GO) biological process enrichment analysis of hemin-induced genes, highlighting pathways related to oxidative stress response, glutathione biosynthesis, and wound healing. **(B)** KEGG pathway enrichment analysis, identifying activation of MAPK, IL-17, and NF-κB signaling pathways. Together, these findings indicate that hemin, as a single agent, preferentially induces a heme-adaptive and cytoprotective transcriptional program, distinct from its selective suppressive effect on interferon-associated gene expression observed under cytokine stimulation.

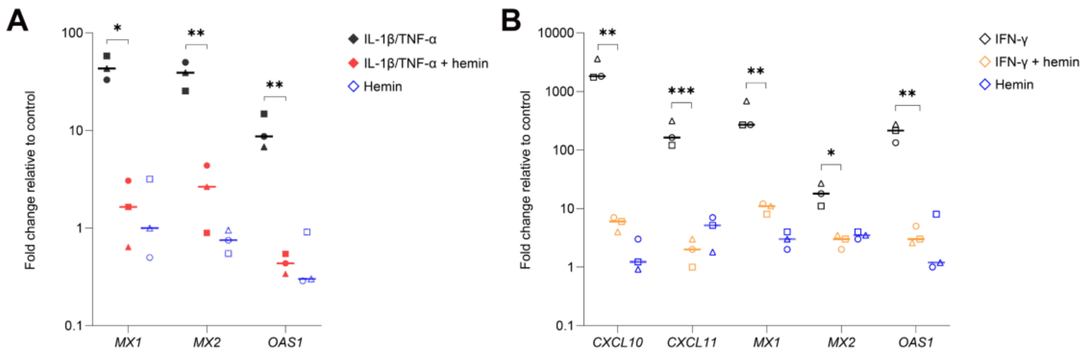

Supplementary Figure 3. Hemin Suppresses Interferon-Associated Gene Expression in Periodontal Cells.

(A) RT-qPCR analysis of classical ISGs (*MX1*, *MX2*, and *OAS1*) mRNA expression in HSC-2 cells stimulated with IL-1β/TNF-α in the presence or absence of hemin. **(B)** RT-qPCR analysis of *CXCL10*, *CXCL11*, *MX1*, *MX2*, and *OAS1* mRNA expression in primary gingival fibroblasts stimulated with IFN-γ in the presence or absence of hemin. Data are presented as fold change relative to untreated controls. Symbols stand for different independent experiments. *$p < 0.05$, **$p < 0.01$, ***$p < 0.001$.

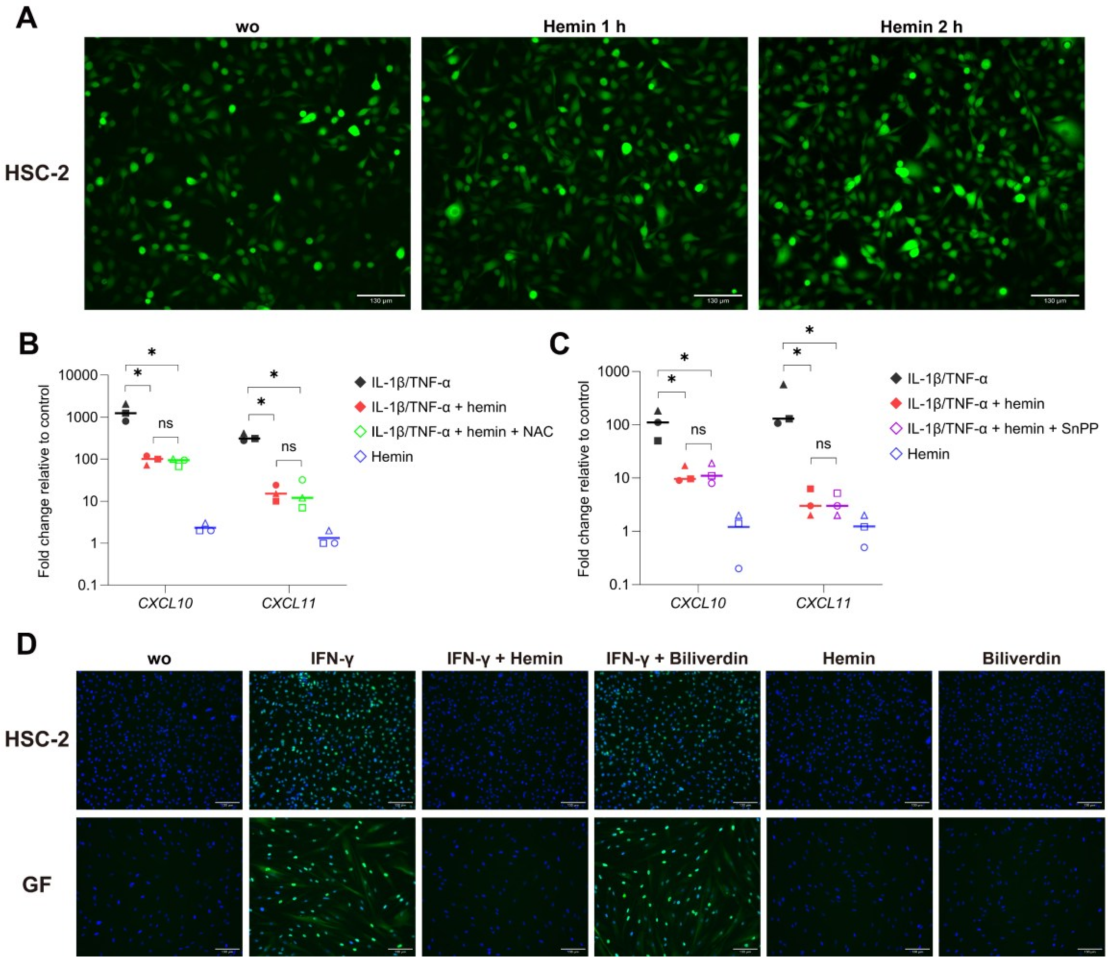

Supplementary Figure 4. Hemin suppresses *CXCL10* and *CXCL11* expression independently of reactive oxygen species and HO-1 catalytic activity in HSC-2 cells.

(A) Representative fluorescence images showed no visually appreciable increase in intracellular ROS levels in HSC-2 cells following hemin treatment for 1 or 2 h compared to untreated controls. Scale bars, 130 μm. **(B)** NAC-mediated scavenging of intracellular ROS did not restore *CXCL10* and *CXCL11* expression, indicating that hemin's suppressive effect is independent of oxidative stress. **(C)** Effect of tin protoporphyrin (SnPP) on hemin-mediated chemokine suppression. Pharmacological blockade of heme oxygenase-1 (HO-1) catalytic activity by SnPP, a competitive inhibitor, failed to rescue *CXCL10* and *CXCL11* expression, demonstrating that hemin acts independently of HO-1 enzymatic function. Symbols stand for different independent experiments. *$p < 0.05$. **(D)** Representative immunofluorescence of phospho-STAT1 in HSC-2 cells and primary gingival fibroblasts (GF), either untreated (wo) or stimulated with IFN-γ alone or in combination with hemin or biliverdin, or treated with hemin or biliverdin alone. Hemin, but not biliverdin, abolished IFN-γ-induced nuclear pSTAT1 accumulation in both cell types. Scale bars, 130 μm. These data suggest hemin suppresses *CXCL10* and *CXCL11* expression by blocking nuclear pSTAT1 accumulation in oral mucosal cells, independent of ROS production and HO-1 activity.

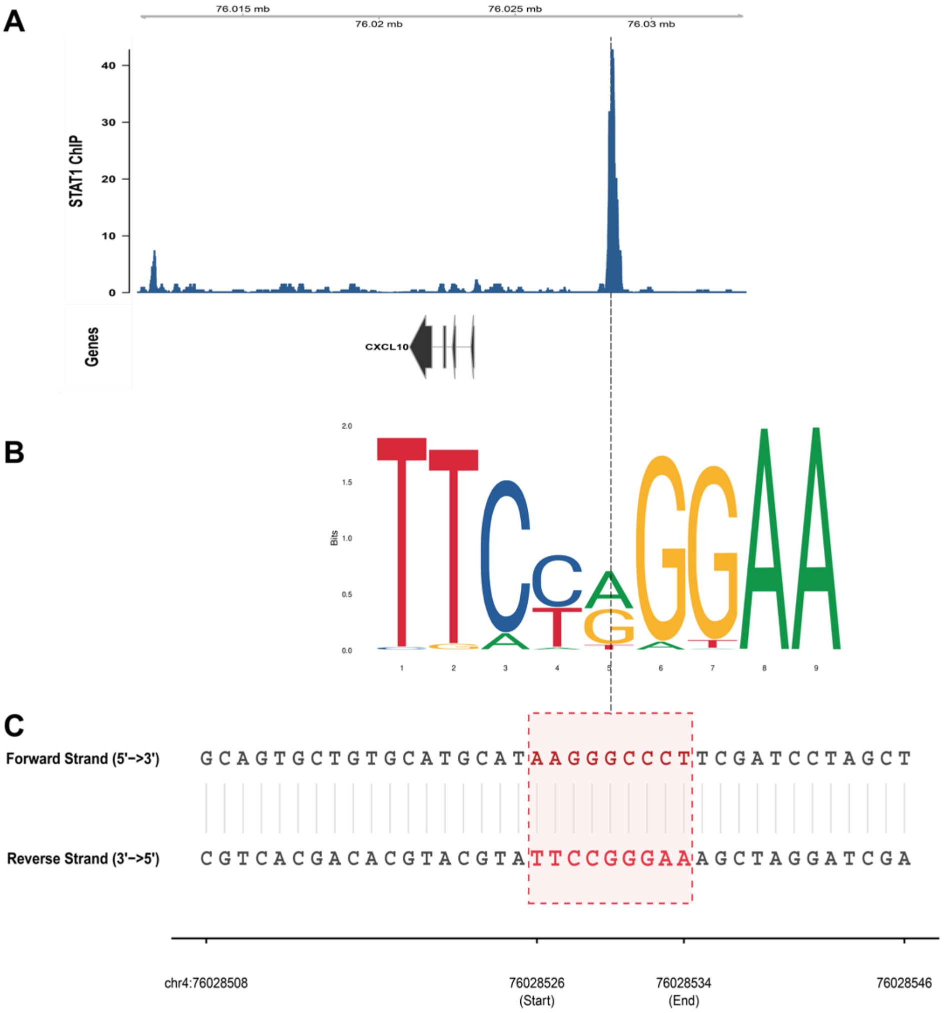

Supplementary Figure 5. STAT1 occupancy at the *CXCL10* locus.

Public STAT1 ChIP-seq signal tracks were visualized across the *CXCL10* locus to provide regulatory context for *CXCL10* as a STAT1-associated gene. **(A)** Genomic track showing STAT1 enrichment across the *CXCL10* regulatory region. **(B)** STAT1-associated sequence motif. **(C)** Genomic sequence surrounding the mapped STAT1-enriched region. These data support the relevance of STAT1 to *CXCL10* regulation but are shown in the Appendix because STAT1 ChIP-seq was not performed after hemin treatment.

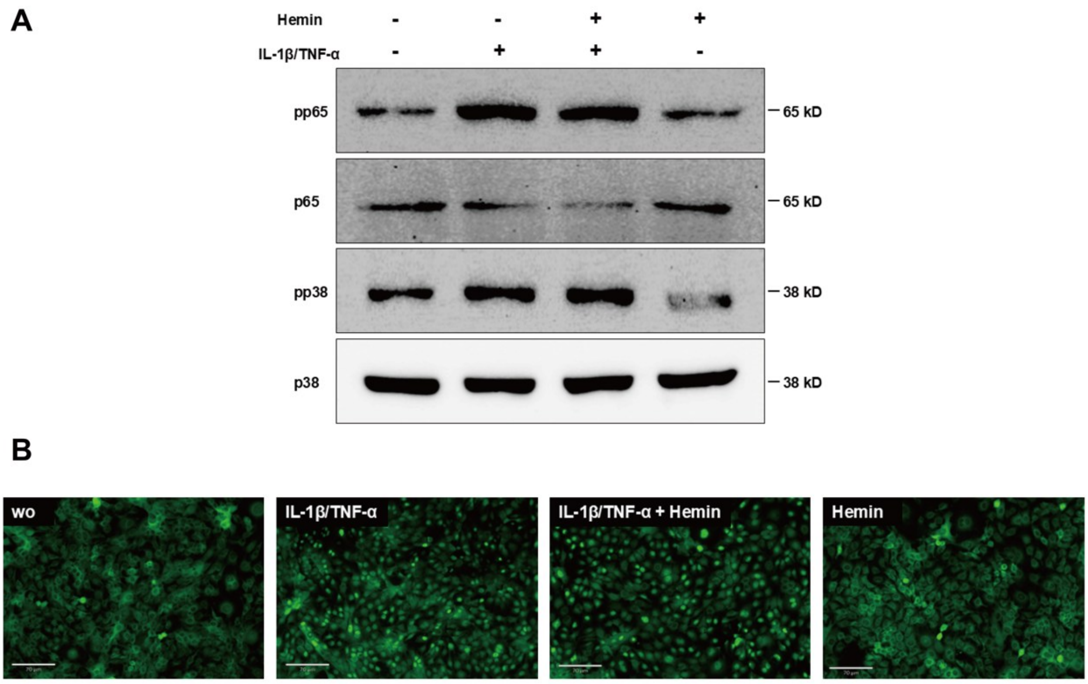

Supplementary Figure 6. Hemin does not globally block NF-κB/MAPK signaling.
HSC-2 cells were exposed to IL-1β/TNF-α for 1 h, either with or without 15-min hemin pretreatment, and parallel inflammatory signaling pathways were assessed. **(A)** Representative Western blot analysis of phosphorylated p65/p38 and total p65/p38. **(B)** Representative immunofluorescence imaging of NF-κB p65 subcellular localization in HSC-2 cells. Serum-free medium alone served as the negative control (wo). The robust nuclear translocation of p65 induced by IL-1β/TNF-α remained strictly unimpeded upon hemin co-treatment, demonstrating its high target specificity. Scale bars, 70 μm. These data support the conclusion that hemin preferentially affects STAT1-dependent signaling rather than producing a generalized blockade of inflammatory signaling.
